# Activity of the *Bacillus anthracis *20 kDa protective antigen component

**DOI:** 10.1186/1471-2334-8-124

**Published:** 2008-09-22

**Authors:** Rasha Hammamieh, Wilson J Ribot, Terry G Abshire, Marti Jett, John Ezzell

**Affiliations:** 1 Walter Reed Army Institute of Research, Silver Spring, Maryland, USA; 2 Diagnostic Systems Division, United States Army Medical Research Institute of Infectious Diseases, Fort Detrick, Frederick, Maryland, USA; 3 Bacteriology Division, United States Army Medical Research Institute of Infectious Diseases, Fort Detrick, Frederick, Maryland, USA

## Abstract

**Background:**

Anthrax is caused by *Bacillus anthracis *that produce two exotoxins, lethal toxin and edema toxin. The lethal toxin is composed of the lethal factor (LF) complexed with the cell binding protective antigen (PA_83_, 83 kDa). Likewise, the edema factor (EF) binds to the PA_83 _to form the edema toxin. Once PA83 is bound to the host cell surface, a furin-like protease cleaves the full-length, inactive protein into 63 kDa and 20 kDa antigens (PA_63 _and PA_20_). PA_63 _forms a heptamer and is internalized via receptor mediated endocytosis forming a protease-stable pore, which allows EF and LF to enter the cell and exert their toxic effects.

Both proteolytically cleaved protective antigens (PA_63 _and PA_20 _fragments) are found in the blood of infected animals. The 63 kDa protective antigen PA_63 _fragment has been thoroughly studied while little is known about the PA_20_.

**Methods:**

In this study we examined the role of PA_20 _using high throughput gene expression analysis of human peripheral blood mononuclear cells (PBMC) exposed to the PA_20_. We constructed a PA mutant in which a Factor Xa proteolytic recognition site was genetically engineered into the protective antigen PA_83 _to obtain PA_20 _using limited digestion of this recombinant PA_83 _with trypsin.

**Results:**

Global gene expression response studies indicated modulation of various immune functions and showed gene patterns indicative of apoptosis via the Fas pathway in a subset of the lymphoid cells. This finding was extended to include observations of increased Caspase-3 enzymatic activity and the identification of increases in the population of apoptotic, but not necrotic cells, based on differential staining methods. We identified a list of ~40 inflammatory mediators and heat-shock proteins that were altered similarly upon exposure of PBMC to either rPA_20 _or *B. anthracis *spores/vegetative cells.

**Conclusion:**

This study shows that the PA_20 _has an effect on human peripheral blood leukocytes and can induce apoptosis in the absence of other PA components.

## Background

*Bacillus anthracis*, the etiologic agent of anthrax, possesses three primary, plasmid-encoded, virulence factors: lethal and edema Toxins encoded by the pXO1 plasmid [1,2] and a poly-γ-D-glutamic acid capsule, encoded by the pXO2 plasmid [1]. Lethal toxin is composed of lethal factor (LF, 90.5 kDa) [3], a Zn^+2 ^dependent metalloprotease which cleaves several members of the mitogen activated protein kinase kinase (MAPKK) family [4-7] and, in complex with protective antigen (PA, 63 kDa referred to as PA_63_), is responsible for the lethal action of anthrax toxin. Similarly, edema toxin is composed of PA_63 _in combination with edema factor (EF, 88.8 kDa), a calmodulin-dependent adenylate cyclase that elevates host target cell intracellular cyclic AMP levels causing deregulation of cellular physiology and edema [8]. Protective antigen is secreted by the organism as an 82.7-kDa protein referred to as PA_83 _[9] and only binds LF or EF when activated by protease cleavage to form PA_63 _[10]. In a model based on studies in cell culture, PA_83 _binds to ubiquitous host cell membrane receptors [11] and is cleaved by a cell-associated furin type protease [12,13] to form PA_63_, which then oligomerizes with other PA_63 _molecules to form an heptamer. The heptamer forms a prepore structure to which LF or EF bind to form lethal toxin or edema toxin, respectively [10]. It has generally been assumed that the 20 kDa remainder of the PA_83 _molecule following cleavage serves no function. Once formed, the complex is translocated into the target host cell where LF and EF exert their toxic effects [14,15]. In contrast to this widely held cell culture model, no PA_83 _has been demonstrated in the peripheral blood of infected animals, but only PA_63 _complexed with LF, and possibly EF, was found thereby supporting the model that the PA_63_/LF complex is pre-formed before binding to the target cell [16,17]. Serum protease activity has been reported that rapidly cleaves PA_83 _to form PA_63 _and rPA_20_. This activity is heat labile at 56°C, requires calcium, and occurs in a broad variety of animals, including primates, horses, bovines, guinea pigs, rabbits, and chickens [16,17]. Our objective was to determine if rPA_20 _has activity on human peripheral blood leukocytes and obtain information as to which type of leukocytes was being affected.

Note: Studies were conducted initially with the commercially prepared 20 kDa fragment of PA, from LIST Biological Laboratories, Inc., which required purification to remove minor amounts of contaminating PA_63_. It is important to preface this report by stating that after a few studies were conducted with the purified PA_63 _free material it was determined that the commercially obtained product was actually 17 kDa rather than 20 kDa. Therefore, we prepared a recombinant (r) PA_20 _that lacked the trypsin cleavage site that would produce PA_17_. Upon trypsin cleavage, the resultant rPA_20 _was purified and used in all subsequent studies. However, similar data were obtained using the List preparation PA_17 _and in-house rPA_20 _for the global microarray studies. All subsequent studies were carried out using only rPA_20_.

Note: microarray data have been submitted to the Gene Expression Omnibus (GEO) and can be searched using the Platform ID: GPL3033, Series:GSE12533.

## Methods

### Removal of PA_63 _from PA_17 _by immunoaffinity

Commercially obtained 20 kDa fragment of PA, (LIST Biological Laboratories, Inc, Campbell, CA) is now referred to as PA_17_. It contained trace amounts of PA_63 _and removal of this contamination was achieved by immunoaffinity chromatography. In this procedure the monoclonal antibody BA-PA2II-14B7-1-1 (1 ml of ascites fluid), was immobilized using an ImmunoPure™ Protein G IgG Orientation Kit (Pierce) as instructed by the manufacturer. This antibody recognizes the 63 kDa receptor binding region (C-terminus) of the PA molecule [18]. PA_17 _kDa N-terminal fragment (LIST Biological Laboratories, Inc.; sold as PA20), 500 μl containing 250 μg of protein, was diluted with an equal volume of PBS (pH 7.3, Sigma, St. Louis, MO). The resulting 1 ml was combined with 1 ml of immobilized antibody and incubated at room temperature on a rotator for 2 hours. The suspension was then centrifuged at 3000 × g for 5 minutes to recover PA_17 _in the supernatant fraction, filtered through a 0.2 μm cellulose acetate low protein binding filter (Corning, Lowell, MA) and frozen in aliquots at -70°C. Proteins were analyzed by SDS PAGE using 4–15% PhastGels (Amersham, Piscataway, NJ) and Western Blot as previously described [16].

### Construction of a PA-Factor Xa recombinant strain and purification of PA 20 kDa fragment designated as rPA_20_

The amino-terminal domain of PA is cleaved at the consensus R164–K165–K166–R167 sequence recognized by furin-like proteases *in-vitro *[12] and by a plasma protease *in vivo *[16]. This process results in the release of a 20-kDa amino-terminal fragment (PA_20_) and the formation of 63-kDa carboxy-terminal fragment heptamers [19]. Lethal factor (LF) and/or edema factor (EF) then bind to the heptamers and these toxic complexes are internalized via receptor-mediated endocytosis into eukaryotic cells [20]. Limited digestion of PA with trypsin results in 63 kDa and 20 kDa fragments. These fragments were isolated and fully characterized by Christensen et al. [21]. However, prolonged digestion with trypsin results in a trypsin resistant 17 kDa amino terminal fragment. Deletion of the consensus R164–K165–K166–R167 sequence eliminates the cleavage of PA by furin-like proteases and by trypsin [22]. *In-vivo *proteolysis of PA results in 63 kDa and 20 kDa fragments [16]; therefore we wished to be able to produce an identical and stable 20 kDa fragment *in-vitro*. In this study, we performed mutagenesis of the trypsin cleavage site in PA83 to make it sensitive to cleavage by Factor Xa protease because there are no other Factor Xa sensitive sites on the PA83 sequence. We constructed a PA mutant in which a Factor Xa proteolytic recognition site (IEGR) was genetically engineered into PA [12]. The Factor Xa proteolytic site was introduced into PA at the trypsin-sensitive site by a 2-step mutagenesis procedure using a Muta-Gene Phagemid *InVitro *Mutagenesis kit (BioRad, Hercules, CA). A 2,044 bp HindIII/BamHI fragment encoding the carboxy-teminus of *pag*, including amino acid residues 164–167 which comprise the trypsin-sensitive site, was inserted into pBluescriptSK (Stratagene, La Jolla, CA.) and designated pPAHB. Oligonucleotide XaFN (5'-GTACTTCGCTTTTCTATTGAGTTCGAAG-3') was used to convert the wild-type *pag *gene fragment in pPAHB to an R164I/K165E double mutant designated pPAHB(XaFN). Oligonucleotide Xa_2_FN_1 _(5'-GTACTTCGCCCTTCTATTGAGTTCGAAG-3') was used to convert the *pag *double mutant in pPAHB(XaFN) to K166G to complete the creation of the Factor Xa site and was designated pPAHB(Xa_2_FN_1_). A 670 bp PstI/HindIII fragment from pPAHB(Xa_2_FN_1_) containing the Factor Xa site codons was ligated into pYS5 similarly digested with PstI/HindIII to remove the wild type 670 bp fragment and the resulting plasmid was designated pYS_1_Xa_2_FN_1 _[22]. pYS_1_Xa_2_FN_1 _was transformed into *Bacillus subtilis *WB600 for expression of the PA/Factor Xa mutant [23]. PA/Factor Xa was purified from WB600 PYS_1_Xa_2_FN_1 _as previously described for rPA [24].

### Endotoxin assay

We carried out endotoxin assays on the LIST PA20 and the Factor Xa PA20 that we prepared. Both preparations had less than 0.1 EU/ml as determined by using the Limulus Amebocyte Lysate (LAL) QCL-1000 assay kit (Cambrex Bio Science Walkersville, MD)

### Exposure of monocytes and lymphocytes to rPA_20_

Leukopheresis units were obtained from volunteer donors using the procedures outlined in our approved human use protocol, reviewed by the established Institutional Review Board at WRAIR. The written informed consent document was provided to the volunteers in advance of the procedure.

We obtained PBMC (4 different individuals over a period of ~6 months, collected from ~8–10 AM to minimize variability) from healthy human male volunteers who had been screened to be HIV and Hepatitis B negative and were from 19–61 years of age.

rPA_20 _was added to newly plated cells in flasks for the time period specified. Cells incubated in the absence and presence of rPA_20 _were collected by centrifugation at the specified exposure time.

### Exposure of monocytes and lymphocytes to the anthrax spores

Spores were prepared from *B. anthracis *Ames strain (pXO1+, pXO2+). Briefly, 5% sheep blood agar (SBA) plates were inoculated with *B. anthracis *Ames spores and incubated overnight at 35°C. Several isolated colonies were transferred to a sterile screw capped tube containing 5 ml of sterile PBS. NSM Petri plates (New sporulation medium: per liter added Tryptone; 3 g, Yeast extract; 3 g, Agar; 2 g, Lab Lemco agar; 23 g, and 1 ml of 1% MnCl_2_·4H_2_O) were inoculated with 200 μl of the prepared cell suspension. The plates were incubated for 48 hrs at 35°C and checked for sporulation progress by microscopic examination. Continued incubation at room temperature was performed until free refractive spores constituted 90–99% of total suspension. Spores were then harvested from plates using 5 ml of sterile water. Spores were washed 4 times in sterile water and checked for purity by plating 10 μl in triplicate onto 5% SBA plates and incubating overnight @ 35°C. Enumerations of spores were calculated via CFU/ml (determination of viable spores) and also for actual spores/ml using Petroff Hauser chamber.

### ELISA immunoassays

An ELISA kit for TNF-α was used to determine TNF-α levels in PBMC cells treated with rPA_20 _according to manufacturer's instructions (Quantikine R&D systems, Minneapolis, MN). The amount of protein was quantified using Ceres UV 900-Hdi plate reader (Bio-Tek Instruments Inc., Winooski, VT).

### Extraction of RNA

Total RNA was isolated from cells using the TRIzol™ reagent (Invitrogen, Carlsbad, CA) according to the manufacturer's instructions. The RNA samples were treated with DNase-1 to remove genomic DNA and were re-precipitated with isopropanol. The quality of the RNA to be used for microarray was characterized using a 2000 BioAnalyzer (Agilent, Santa Clara, CA) to verify the presence of 18 and 28S bands, to confirm the lack of degradation. RNA quantity was determined using a Nanodrop spectrophotometer.

### cDNA microarrays

Preparation of Microarray Chip: Human cDNA microarrays were generated using sequence verified PCR elements including the approximate 6900 well-characterized human genes from The Easy to Spot Human UniGEM V2.0 cDNA (Incyte Genomics, Inc). The PCR products ranging from 500–700 base pairs were deposited in 3X saline sodium citrate (SSC) at an average concentration of 165 μg/μl on CMT-GAPS II aminopropyl silane-coated slides (Corning, Corning, NY) using a VersArray microarryer (Bio-Rad, Inc). The arrays were post processed by UV-cross linking at 1200 mjoules, baking for 4 hours at 80°C, and then the positively charged amine groups on slide surface were inactivated through reacting with succinic anhydride/N-methyl-2-pyrrolidinone. Upon hybridization, the quality of each microarray, i.e. the efficiency of reverse transcription (RT) reactions, labeling competence etc. was assessed.

### Microarray hybridization and image processing

Microarray labeling was performed using Micromax Tyramide Signal Amplification (TSA) Labeling and Detection Kit (Perkin Elmer, Inc., MA). The slides were hybridized for 16 h at 60°C. The GenePix Pro 4000b (Axon Instruments, Inc., CA) optical scanner was used to scan the hybridized slides and the raw intensity was recorded through the Gene Pix 4000 software package (Axon Instruments, Inc., CA). Intensity of the scanned images was digitalized through Genepix 4.0 software.

### Data analysis

Assessment of the overall integrity of the microarray experiment:

Microarray images were visualized using Imagene v.6 (BioDiscovery, Inc., El Segundo, CA) and data were analyzed using GeneSpring V. 7.1 (Agilent, Santa Clara, CA) and Partek Pro. V. 5.0 (Partek, St Louis, MI). Data cleansing and normalization: Using ImaGene (BioDiscovery Inc), background and foreground pixels of each spot were segmented and the highest and lowest 2% of the probe intensity was discarded. Local background correction was applied to each individual spot. The genes that passed this filter in all given experiments were selected for further study.

Data cleansing and statistical analysis was carried out using GeneSpring^® ^7.1 (Agilent Tech., CA). Local background was subtracted from individual spot intensity. Genes that failed this 'background check' in any of the experiments were eliminated from further analysis. Each chip was next subjected to intra-chip normalization (LOWESS). The genes that varied most between control and treated sample sets were selected via *t*-test analysis. The *p*-value cutoff was set at 0.05.

We used the reference design, where a reference RNA sample is co-hybridized with each sample on the slide. This design allows us to normalize between slides for variations that can be due to hybridization, transcription and labeling efficiencies (technical variations).

### Apoptosis study using Hoechst 33258

PBMC were treated with rPA_20 _for 24 h. Cells were stained with Hoechst 33258 dye for 30 min and examined by fluorescence microscopy. Cells having bright; fragmented and condensed nuclei were identified as apoptotic cells. The number of apoptotic cells was counted in 10 microscopic fields (×40) in each case.

### Caspase enzymatic assay

Caspase activity in PBMC cells exposed to LIST PA_17 _and to rPA_20 _was studied using the EnzChek^® ^Caspase-3 Assay Kit #2 (Invitrogen, Carlsbad, CA). Cells were harvested after 24 hrs of exposure to rPA_20 _and washed in PBS. Cells were lysed and centrifuged. The Z-DEVD-R110 substrate solution was added to each of the treated and control samples. The mixture was incubated for 30 min and the fluorescence was measured at excitation/emission ~496/520 nm.

### CD38 staining of PBMC cells

Peripheral blood mononuclear cells were incubated with rPA_20 _for 16 hours. Cells were then washed twice with PBS and labeled with allophycocyanin (APC)-conjugated mouse anti-human CD38 monoclonal antibody (Becton Dickinson Biosciences, Franklin Lakes, NJ), followed by incubation on ice for 30 min in the dark. The cells were then washed and resuspended at 2 × 10^6 ^cells/ml in cell buffer (cell assay reagents, Agilent Technologies, Palo Alto, CA). A cell assay LabChip (Agilent Technologies) was primed with priming solution (Agilent Technologies), after which 10 μl of the cell suspension (20,000 cells) was added to one of six channels. A focusing dye was applied to another chamber, which acted as a reference for the optical detection system. The chip was then placed in an Agilent Technologies Model 2100 bioanalyzer and fluorescence from the cells was measured. Fluorescent events were plotted against the fluorescent intensity (frequency histogram).

## Results

### Analysis of PA_17 _obtained from LIST Biologicals, Inc

**Figure 1:**
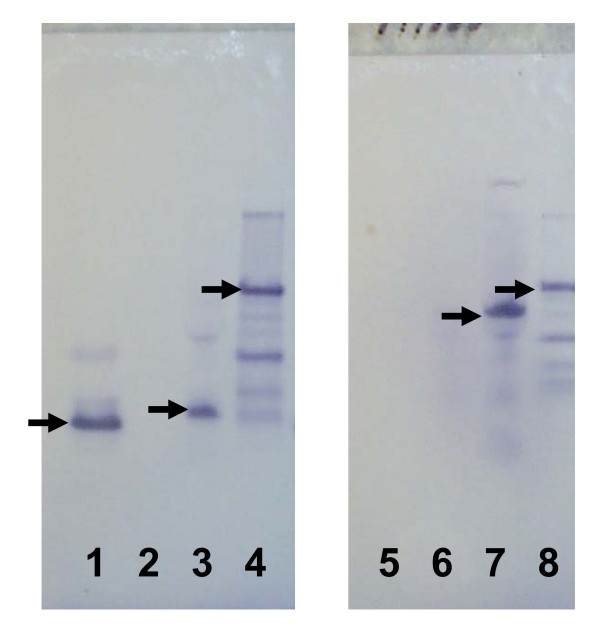
**SDS-PAGE Western blot of anthrax infected rabbit plasma showing PA_20 _and PA_63 _just prior to death.** Lanes 1 to 4 were stained with anti-PA_20 _specific MAb and lanes 5 to 8 stained with anti – PA63 specific MAb. Lanes 1 (arrow points to PA_17_) and 5 were loaded with the LIST PA_17 _at 5 mg/ml. Lanes 2 and 6 were left blank to minimize cross contamination. Lanes 3 (arrow points to PA_20_) and 7 (arrow points to PA_63_) were loaded with 1:20 dilution of plasma from Ames strain challenged rabbit #41 just prior to death. Lanes 4 (arrow points to PA_83_) and 8 (arrow points to PA_83_) were loaded with purified PA83 @ 10 mg/ml.

Subsequent to portions of this investigation, it was noted in other studies that the PA fragment obtained from LIST Biologicals, Inc., appeared smaller on Western blots than PA_20 _detected in blood of infected animals (Figure 1). As can be seen in lane 1 the purported 20 kDa PA from LIST was smaller than the PA_20 _from infected animals (lane 3). To address this disparity in size, Dr. Harry Hines and his staff in the Toxinology Division, USAMRIID, performed electrospray mass spectrometry on the commercially obtained PA_17_. It was determined that the size of the protein moiety was 17 kDa rather than 20 kDa. Upon this discovery, an alternative source of PA_20 _was generated through recombinant DNA methodology as described in the following section.

### Construction of a PA-Factor Xa recombinant strain and purification of PA 20 kDa fragment designated as rPA_20_

**Figure 2:**
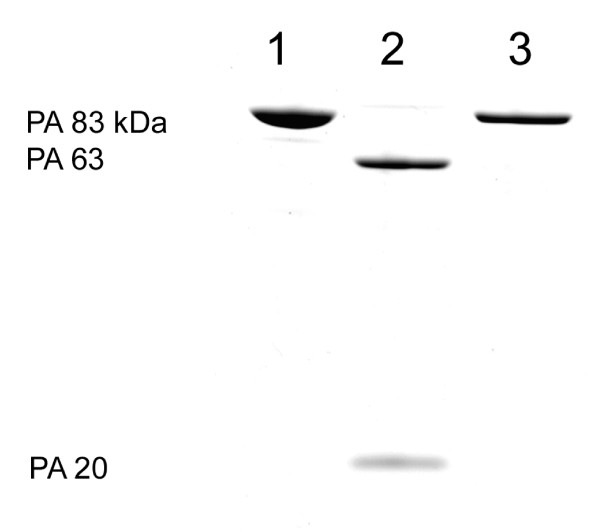
**SDS-Page analysis of the _83 _and the PA_63 _and PA_20 _fragments. PA/Factor Xa was purified from WB600 PYS1Xa2FN1 (Lane 1).** The purified protein was digested with Factor Xa protease (Lane 2) resulting in PA63 and PA20 fragments; Wild type control PA83 treated with Factor Xa protease did not result in PA fragments (Lane 3).

The two furin-like protease or trypsin cleavage sites in PA result in a 20 kDa fragment that subsequently is reduced to 17 kDa. In order to prevent that from occurring, the sequence R164–R167 was changed from RKKR to IEGR as described in materials and methods. Deletion of the consensus R164–K165–K166–R167 sequence was shown previously to eliminate the cleavage of PA by furin-like proteases and by trypsin [22]. The purified protein was digested with Factor Xa protease (Figure 2). SDS PAGE and N-terminal sequence analysis confirmed that the 63-kDa and 20 kDa fragments produced were identical to the fragments produced from wild type PA by limited trypsin digestion (not shown).

### Purification of the 20 kDa fragment from PA after insertion of the Factor Xa sensitive sequence

Purified 83 kDa rPA was treated for 30 min at 37°C with bovine plasma Factor Xa (Pierce) resulting in PA 20 and 63 kDa fragments. LF was added and allowed to oligomerize with the PA_63 _at room temperature for 15 minutes. The mixture was applied to Superose 6 size exclusion column (Amersham-Pharmacia) in PBS and resulted in 3 peaks. The proteins in each peak were identified by SDS PAGE and Western blot. The first peak contained PA_63 _and LF, the second peak contained LF and the third peak contained rPA_20_. The rPA_20 _peak was further purified by Immunoaffinity to be certain that the rPA_20 _would not contain any residual PA_63 _as follows.

**Figure 3:**
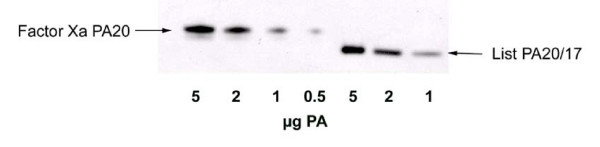
**Western blot comparison of recombinant PA_20 _from the PA/Factor Xa mutant (lanes 1–4) and the commercial PA_17 _(lanes 5–7) product at quantities varying from 1 to 5 μg of the purified rPA_20 _Factor Xa and the LIST PA_17_. **Purified 83 kDa PA/Factor Xa was treated for 30 min at 37°C with bovine plasma Factor Xa resulting in PA_20 _and PA_63 _kDa fragments. LF was added and allowed to oligomerize with the PA_63 _at room temperature for 15 minutes. The mixture was applied to Superose 6 size exclusion column (Amersham-Pharmacia) in PBS and resulted in 3 peaks. The rPA_20 _was further purified by Immuno-affinity.

The monoclonal antibody BA-PA2II-14B7-1-1 (1 ml of ascites fluid) which recognizes the receptor binding region (C-terminus) of the PA molecule was immobilized using an ImmunoPure™ Protein G IgG Orientation Kit (Pierce) as instructed by the manufacturer [18]. 200 μg of rPA_20 _kDa (n-terminal PA fragment purified by the Superose exclusion column above) was combined with 1 ml of immobilized antibody and incubated at room temperature on a rotator for 2 hours. The suspension was then centrifuged at 3000 × g for 5 minutes to recover the rPA_20 _containing supernatant fraction. The purified rPA_20 _was compared to the LIST PA_17 _by SDS PAGE using 4–15% PhastGels (Amersham) (Figure 3).

### Gene expression patterns of PBMC exposed to rPA_20 _in vitro

**Figure 4:**
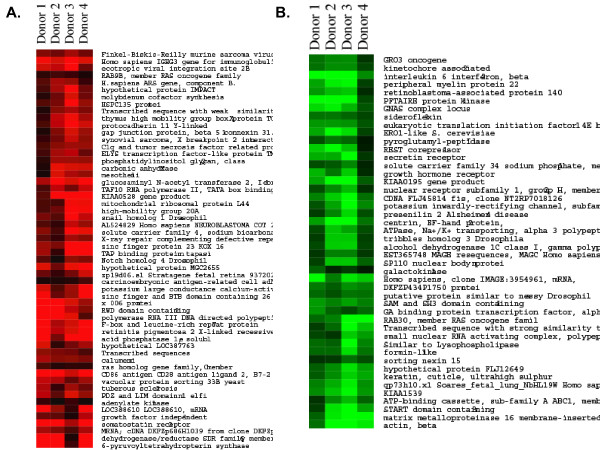
**Pseudo color cluster view of up regulated (a) and down regulated genes (b) in PBMC in response to 2 μg/ml the rPA_20_. **Cells were obtained from 4 different donors and were treated with the rPA_20_. RNA was isolated and hybridized on the cDNA microarray slides as detailed in Materials and Methods. Images were analyzed using GenePix 4.0 and data were analyzed using GeneSpring 7.0. The expression data for these genes are listed in table 1. These genes were identified, using ANOVA t-test with a p < 0.05, to be highly significantly regulated when compared to the control untreated cells.

PBMC samples obtained from 4 healthy individuals were incubated with 2 μg/ml of the rPA_20 _for 4 hrs. Microarray experiments were carried out using custom made cDNA chips. The RNA quality was characterized beforehand using a BioAnalyzer 2000 (Agilent, CA). Upon hybridization, the quality of each microarray, i.e. the efficiency of reverse transcription (RT) reaction, labeling competence, were assessed using RNA spikes (Invitrogen, CA). Inter-chip and intra-chip data normalizations were computed, as described in the Materials and Methods, using GeneSpring Software (Silicon Genetics, CA). One-way ANOVA with a p-value < 0.05 was applied to identify genes differentially regulated by rPA_20_. Figures 4a and 4b are cluster views of gene expression profiles in PBMC cells obtained from four donors and exposed to rPA_20_.

**Table 1:** Gene identified to be highly significantly regulated by *B. anthracis*

**Gene/protein**	**Donor 1**	**Donor 2**	**Donor 3**	**Donor 4**
6-pyruvoyltetrahydropterin synthase	2.265	0.4394	2.262	2.789
acid phosphatase 1, soluble	1.845	2.053	0.8392	3.292
actin, beta	-1.667	-2.469	-1.813	-0.7812
adenylate kinase 1	0.1362	0.4168	0.1217	0.406
alcohol dehydrogenase 1C (class I), gamma polypeptide	-0.4945	-1.479	-1.452	-0.522
associated molecule with the SH3 domain of STAM (AMSH) like pro	-1.286	-0.7072	-1.122	-0.319
AT rich interactive domain 5A (MRF1-like)	-1.117	-1.173	-0.608	-0.7124
ATPase, aminophospholipid transporter (APLT), Class I, type 8A, m	-0.867	-0.9902	-1.217	-1.002
ATPase, Ca++ transporting, plasma membrane 2	-1.185	-0.8227	-1.454	-1.233
ATPase, H+ transporting, lysosomal 38 kDa, V0 subunit d isoform 1	-1.254	-0.7821	-0.7238	-0.1514
ATPase, Na+/K+ transporting, alpha 3 polypeptide	-0.5454	-1.172	-1.408	-0.5249
ATP-binding cassette, sub-family A (ABC1), member 3	-0.7075	-1.336	-0.9859	-0.3627
beta-transducin repeat containing	-1.301	-1.458	-1.57	-0.5347
bromodomain and PHD finger containing, 3	-0.6459	-0.2634	-1.069	-0.3741
C1q and tumor necrosis factor related protein 1	1.072	1.119	0.9826	0.543
calsyntenin 3	-0.9132	-0.3774	-1.219	-0.3527
calumenin	0.8976	1.009	0.984	1.217
carbonic anhydrase X	1.494	1.533	1.664	0.2302
carcinoembryonic antigen-related cell adhesion molecule 7	0.5028	0.263	0.2265	0.2129
cathepsin D (lysosomal aspartyl protease)	-0.4152	-0.3937	-1.185	-0.9571
CD86 antigen (CD28 antigen ligand 2, B7-2 antigen)	1.8	1.456	2.398	2.843
CDNA FLJ45814 fis, clone NT2RP7018126	-0.7894	-1.97	-1.454	-0.923
centrin, EF-hand protein, 2	-0.8196	-2.365	-2.736	-1.134
chemokine (C-C motif) receptor 6	-0.4528	-0.2879	-0.6378	-0.2152
Clone IMAGE:4838790, mRNA	-0.485	-0.9604	-0.515	-0.2199
cofactor required for Sp1 transcriptional activation, subunit 9, 33 kDa	-0.8153	-0.4039	-1.577	-0.6495
complement component 3a receptor 1	-0.7288	-1.094	-1.152	-0.2907
dehydrogenase/reductase (SDR family) member 6	2.449	0.2412	2.836	2.234
DKFZP434P1750 protein	-0.9027	-1.021	-0.6581	-0.8601
dual-specificity tyrosine-(Y)-phosphorylation regulated kinase 2	-0.7681	-1.55	-0.7539	-0.2911
ecotropic viral integration site 2B	0.7891	1.913	1.367	1.481
ELYS transcription factor-like protein TMBS62	0.7329	1.34	1.167	0.4772
endothelin receptor type B	-0.7971	-0.6776	-1.425	-0.9299
ERO1-like (S. cerevisiae)	-0.4751	-1.573	-2.013	-1.192
EST365748 MAGE resequences, MAGC Homo sapiens cDNA, mR	-0.3974	-0.9129	-0.9105	-0.3948
eukaryotic translation initiation factor 4E binding protein 1	-0.6047	-1.043	-1.089	-1.082
family with sequence similarity 20, member A	-0.26	-0.5113	-1.164	-1.137
F-box and leucine-rich repeat protein 7	2.838	1.484	1.225	3.888
fibronectin 1	-0.5673	-0.5845	-1.167	-1.708
Finkel-Biskis-Reilly murine sarcoma virus (FBR-MuSV) ubiquitously	0.7983	1.141	0.7866	0.9328
FLJ20793 protein	-0.9689	-0.9124	-1.154	-0.2331
formin-like 1	-0.8158	-1.599	-0.7554	-0.7411
GA binding protein transcription factor, alpha subunit 60 kDa	-0.7418	-0.5199	-0.7053	-1.07
galactokinase 1	-0.1951	-0.4303	-0.27	-0.6901
gap junction protein, beta 5 (connexin 31.1)	1.095	1.524	0.9336	0.2265
GLE1 RNA export mediator-like (yeast)	-0.8643	-0.341	-0.2584	-0.6748
glucosaminyl (N-acetyl) transferase 2, I-branching enzyme	2.377	1.534	2.844	1.225
GNAS complex locus	-0.6085	-1.294	-1.715	-1.55
GRO3 oncogene	-0.3814	-1.003	-0.9765	-0.7961
growth factor independent 1	1.282	0.2066	0.669	0.7891
growth hormone receptor	-0.3103	-1.458	-0.8417	-1.296
H. sapiens ARS gene, component B.	0.3962	1.059	1.186	0.6116
heat shock 27 kDa protein 2	-0.6707	-0.7274	-2.224	-1.424
hemoglobin, zeta	-0.4909	-0.8871	-0.4189	-0.149
high-mobility group 20A	1.768	2.038	0.7883	0.5271
Homo sapiens IGHG3 gene for immunoglobulin heavy chain gamma	1.585	1.946	1.511	1.761
Homo sapiens, clone IMAGE:3954961, mRNA,	-1.437	-1.642	-0.8906	-1.996
HSPC135 protein	1.058	1.879	0.8552	0.5993
Human DNA sequence from clone XX-D88L2 on chromosome 1q32	-0.9986	-0.4828	-0.7607	-0.2225
hydroxysteroid (17-beta) dehydrogenase 2	-0.6315	-0.6454	-1.138	-0.4903
hypothetical LOC387763	1.165	0.827	0.3265	1.563
hypothetical protein FLJ10925	-1.163	-0.301	-1.229	-1.046
hypothetical protein FLJ12649	-1.138	-1.602	-1.002	-0.6636
hypothetical protein FLJ31842	-0.7517	-0.2782	-0.436	-0.1725
hypothetical protein IMPACT	0.7924	2.146	1.185	0.512
hypothetical protein LOC51321	-1.431	-0.7981	-0.5971	-0.5105
hypothetical protein MGC2655	3.033	1.669	0.7908	1.584
integrin, beta 2 (antigen CD18 (p95), lymphocyte function-associate	-1.358	-1.034	-0.7394	-0.4935
interleukin 6 (interferon, beta 2)	-0.9922	-2.6	-1.892	-1.956
kallikrein 10	-0.7654	-0.3185	-1.353	-0.901
keratin, cuticle, ultrahigh sulphur 1	-1.208	-1.486	-1.002	-0.6479
KIAA0195 gene product	-0.654	-2.765	-1.988	-1.192
KIAA0196 gene product	-1.305	-1.098	-0.8417	-0.8635
KIAA0528 gene product	4.307	2.866	2.187	0.6229
KIAA0759	-0.8301	-0.759	-0.9988	-0.5301
KIAA1458 protein	-1.232	-1.82	-0.9504	-0.4043
KIAA1539	-1.079	-1.772	-1.397	-0.9942
kinesin family member 4A	-1.135	-1.455	-0.8039	-0.2817
kinetochore associated 2	-0.4972	-1.001	-1.011	-0.7954
kruppel-like zinc finger protein; Homo sapiens promyelocytic leukem	-1.251	-0.9379	-1.973	-1.031
kynurenine 3-monooxygenase (kynurenine 3-hydroxylase)	-0.5685	-1.132	-1.798	-0.8039
LOC388610 (LOC388610), mRNA	2.319	0.3752	1.057	2.013
male sterility domain containing 1	-0.6454	-0.5462	-1.525	-1.041
matrix metalloproteinase 16 (membrane-inserted)	-1.777	-2.344	-1.65	-0.6869
mesothelin	1.045	1.22	1.54	0.1712
methylthioadenosine phosphorylase	-1.51	-0.4183	-1.556	-1.119
mitochondrial ribosomal protein L44	1.706	1.743	0.4926	0.544
molybdenum cofactor synthesis 3	0.779	1.529	0.7891	0.2762
MRNA; cDNA DKFZp686H1039 (from clone DKFZp686H1039)	1.206	0.519	0.9084	1.165
mucin 5, subtype B, tracheobronchial	-0.5146	-0.1101	-0.5376	-0.7425
Notch homolog 4 (Drosophila)	0.9252	1.024	0.1454	0.6285
nuclear receptor subfamily 1, group H, member 2	-0.3397	-1.337	-0.9731	-0.5515
paired box gene 3 (Waardenburg syndrome	-0.6751	-0.4723	-0.6409	-0.6302
paraneoplastic antigen MA1	-0.67	-1.225	-0.48	-0.2581
PDZ and LIM domain 1 (elfin)	1.041	1.555	0.6517	1.342
peripheral myelin protein 22	-0.4552	-1.767	-1.706	-1.176
PFTAIRE protein kinase 1	-0.5164	-2	-2.035	-1.931
phosphatidylinositol glycan, class Q	1.067	1.114	1.141	0.3068
phytanoyl-CoA hydroxylase interacting protein	-1.698	-0.07415	-1.755	-1.699
PlSC domain containing hypothetical protein	-1.719	-0.4816	-1.777	-1.5
polymerase (RNA) III (DNA directed) polypeptide G (32 kD)	0.5734	1.622	3.274	3.359
POM (POM121 homolog, rat) and ZP3 fusion	-0.647	-0.319	-0.7233	-0.117
potassium inwardly-rectifying channel, subfamily J, member 15	-0.9743	-2.059	-1.175	-0.5925
potassium large conductance calcium-activated channel, subfamily	2.751	1.112	0.5753	1.485
presenilin 2 (Alzheimer disease 4)	-0.5609	-1.55	-0.9509	-0.4208
proline-serine-threonine phosphatase interacting protein 1	-0.2379	-0.5739	-0.8672	-0.4775
ProSAPiP2 protein	-0.6497	-0.1215	-0.5855	-0.3364
protocadherin 11 Y-linked	1.474	1.825	1.35	0.5331
putative nucleic acid binding protein RY-1	-1.409	-1.09	-0.5389	-0.4293
putative protein similar to nessy (Drosophila)	-0.7926	-0.8206	-0.5677	-0.7377
pyroglutamyl-peptidase I	-0.1691	-0.7233	-0.8544	-0.5551
qp73h10.x1 Soares_fetal_lung_NbHL19W Homo sapiens cDNA clo	-1.024	-1.686	-1.235	-0.9119
RAB30, member RAS oncogene family	-1.838	-2.618	-0.917	-0.7415
RAB9B, member RAS oncogene family	0.2029	0.4147	0.4782	0.3173
RA-regulated nuclear matrix-associated protein	-0.9885	-0.276	-0.4191	-0.4208
ras homolog gene family, member C	0.3334	0.346	0.2774	0.5479
REST corepressor 1	-0.2084	-1.375	-2.173	-1.47
retinitis pigmentosa 2 (X-linked recessive)	2.301	1.546	1.339	3.789
retinoblastoma-associated protein 140	-0.3049	-1.424	-1.341	-1.09
retinol binding protein 4, plasma	-0.6962	-1.256	-2.234	-1.568
ribosomal protein L23	-1.188	-1.077	-1.345	-0.9045
ribosomal protein S20	-2.379	-2.005	-0.4064	-1.469
RNA (guanine-7-) methyltransferase	-0.9669	-0.8633	-1.674	-0.3241
RWD domain containing 2	0.9411	1.362	2.293	2.374
SAM and SH3 domain containing 1	-0.9048	-1.158	-0.9225	-1.106
secretin receptor	-0.3857	-2.202	-1.223	-1.245
semenogelin II	-0.9479	-0.872	-0.5137	-0.1655
SET translocation (myeloid leukemia-associated)	-1.673	-0.5829	-1.633	-0.8802
sideroflexin 1	-0.4931	-0.7209	-0.8337	-0.8135
similar to human GTPase-activating protein	-2.076	-1.635	-1.364	-0.8021
Similar to Lysophospholipase	-0.9395	-1.975	-0.6778	-0.7481
small nuclear RNA activating complex, polypeptide 5, 19 kDa	-1.124	-1.438	-0.372	-0.4988
snail homolog 1 (Drosophila)	1.386	2.22	0.6079	0.8456
solute carrier family 16 (monocarboxylic acid transporters), member	-0.8285	-0.8079	-1.113	-0.09096
solute carrier family 34 (sodium phosphate), member 1	-0.2497	-0.9574	-0.6233	-0.55
solute carrier family 4, sodium bicarbonate cotransporter, member 7	1.666	2.241	0.4895	0.9819
somatostatin receptor 2	3.287	1.207	2.095	2.374
sorting nexin 15	-0.5503	-0.7774	-0.4106	-0.3535
SP110 nuclear body protein	-0.699	-1.42	-1.406	-0.4606
START domain containing 3	-1.17	-2.012	-1.621	-0.4317
suppressor of var1, 3-like 1 (S. cerevisiae)	-1.02	-0.8975	-0.4765	-0.2837
synovial sarcoma, X breakpoint 2 interacting protein	0.9957	1.435	0.8189	0.3763
TAF10 RNA polymerase II, TATA box binding protein (TBP)-associa	1.408	0.8041	1.358	0.5028
TAP binding protein (tapasin)	2.518	1.909	0.3718	1.161
thymus high mobility group box protein TOX	1.165	1.726	1.373	0.3885
toll-like receptor 3	-0.8173	-1.102	-1.35	-0.3895
TRAF and TNF receptor associated protein	-1.192	-1.042	-0.7728	-0.1867
Transcribed sequence with strong similarity to protein ref:NP_05761	-1.382	-2.008	-0.7379	-0.4367
Transcribed sequence with weak similarity to protein ref:NP_06031	0.8875	1.208	0.9568	0.2339
Transcribed sequence with weak similarity to protein sp:P39193 (H.	-1.096	-1.454	-1.585	-0.1049
Transcribed sequences	0.5241	0.8148	0.8891	1.009
Transcribed sequences	-3.77	-2.45	-1.731	-0.6047
tribbles homolog 3 (Drosophila)	-0.4489	-1.411	-1.424	-0.4251
troponin I, skeletal, slow	-0.2777	-0.7372	-1.637	-1.618
tuberous sclerosis 2	0.7672	1.119	0.3896	1.254
tyrosine 3-monooxygenase/tryptophan 5-monooxygenase activation	-1.402	-1.306	-1.059	-0.2737
upstream regulatory element binding protein 1	-1.067	-1.853	-0.8107	-0.3799
vacuolar protein sorting 33B (yeast)	1.124	0.6897	1.236	1.376
WD and tetratricopeptide repeats 1	-1.529	-0.1551	-1.397	-0.9977
x 006 protein	1.105	1.55	3.65	2.442
X-ray repair complementing defective repair in Chinese hamster cell	0.9716	1.156	0.3796	0.6554
zinc finger and BTB domain containing 26	2.651	1.075	0.7553	1.785
zinc finger protein 23 (KOX 16)	2.372	2.401	0.8906	1.1
zinc finger, DHHC domain containing 18	-0.189	-0.3015	-0.6595	-0.228
zp19d06.s1 Stratagene fetal retina 937202 Homo sapiens cDNA clo	1.479	0.8278	0.384	0.7579

**Figure 5:**
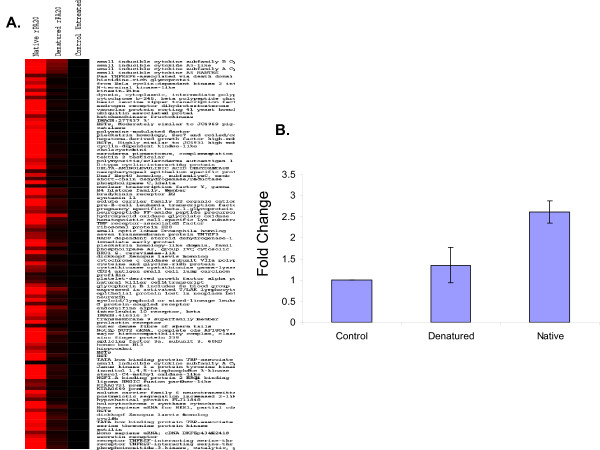
**A. Cluster view of gene expression profiles in PBMC exposed to 2 μg/ml native and denatured rPA_20 _compared to the control untreated.** The peptide was heat denatured at 95°C for 15 min. prior to adding it to the cells. Equal amounts of the native and denatured peptides were added to the cells and incubated for 4 hrs. Data were normalized to the control untreated cells. B. Expression levels of TNF-α in PBMC cells exposed to the native and denatured rPA_20_. Cells were incubated with 2 μg/ml of the two peptides separately and the expression of TNF-α was examined using ELISA.

To confirm that the results observed in PBMC cells exposed to rPA_20 _were effects specific to rPA_20_, we denatured rPA_20 _by heating the peptide at 95°C for 10 min before adding it to the cells. Equal amounts (2 μg/ml) of the native and the denatured peptides were added to the cells and global gene expression analyses were carried out to compare the effect of native and denatured rPA_20 _on PBMC cells (Figure 5a). We found minimal variation in gene expression profiles in PBMC cell exposed to rPA_20 _when compared with the control untreated PBMC (Figure 5b).

We also studied the expression of TNF-α using ELISA in PBMC cells exposed to the native and denatured rPA_20 _and found no significant change in the expression of TNF-α in cells exposed to heat denatured rPA_20_. However, an increase in the expression of TNF-α was observed in cells treated with the native rPA_20 _peptide (Figure 5b).

### Study of the gene expression profiles in PBMC exposed to rPA_20 _compared to *B. anthracis*

**Figure 6:**
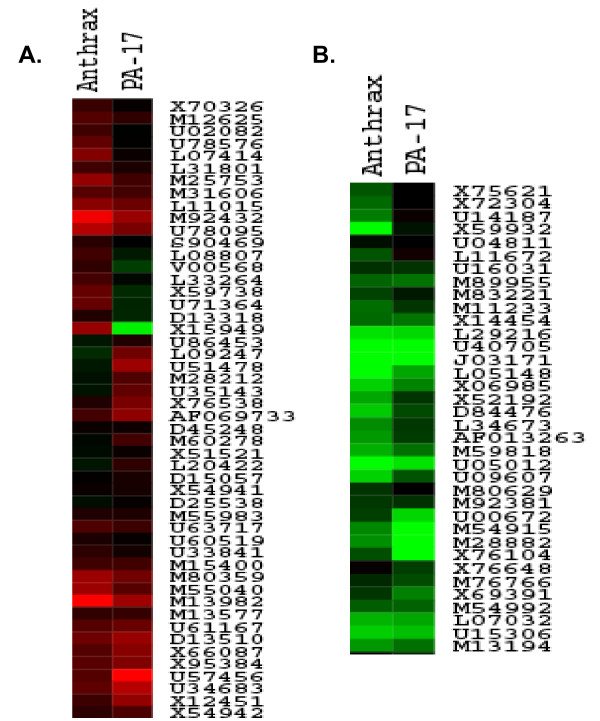
**Cluster analysis of the gene expression profiles of PBMC cells exposed to rPA_20 _compared with those obtained from PBMC exposed to *B. anthracis *for 4 hrs.** RNA samples were isolated and hybridized on the cDNA micorarray slides as detailed in materials and methods. Images were analyzed using GenePix 4.0 and data were analyzed using GeneSpring 7.0 to identify up regulated (6a) and down regulated genes (6b).

The gene expression profiles of PBMC cells exposed to rPA_20 _were compared with those obtained from PBMC exposed to *B. anthracis *at the same time points. We found that a significant number of genes were similarly regulated in rPA_20 _exposed cells when compared to cells exposed to the full pathogen (Figure 6).

### Analysis of genes regulated in response to the rPA_20_

**Figure 7:**
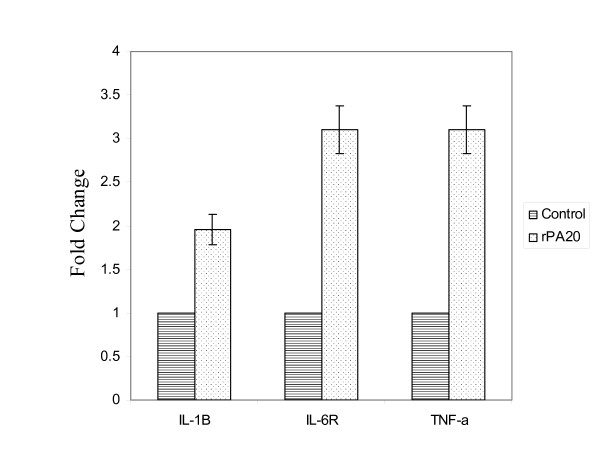
**Expression profiles of IL-1β, IL-6R and TNF-α in response to rPA_20 _in PBMC.** Cells were incubated with the rPA_20 _for 4 hrs. RNA samples were isolated and hybridized on the cDNA micorarray slides as detailed in materials and methods. Images were analyzed using GenePix 4.0 and data were analyzed using GeneSpring 7.0.

We applied ANOVA to identify the statistically significant genes that were altered with a p-value < 0.05 within the control and treated samples. Of the genes that were significantly up regulated by rPA_20 _in PBMC, we identified genes involved in cell adhesion, cell apoptosis, signaling and immune and inflammatory responses. Cytokine related genes were also regulated by rPA_20_. TNF-α, IL-1B, and IL-6 receptor were highly up regulated in PBMC treated with rPA_20 _(Figure 7).

**Figure 8:**
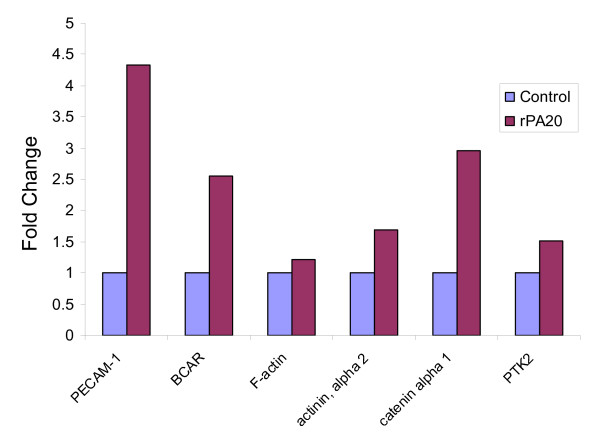
**Expression profile of genes related to the cell adhesion pathway that were up regulated by rPA_20 _in PBMC.** Cells were incubated with the rPA_20 _for 4 hrs. RNA samples were isolated and hybridized on the cDNA micorarray slides as detailed in materials and methods. Images were analyzed using GenePix 4.0 and data were analyzed using GeneSpring 7.0. Data were then analyzed using GeneCite to identify pathways regulated by the rPA_20_. These genes are platelet/endothelial cell adhesion molecule 1 (PECAM1), breast cancer anti-estrogen resistance (BCAR), capping protein (actin filament) muscle Z-line, alpha 1 (F-actin), actinin, alpha 2, catenin alpha and PTK2 protein tyrosine kinase 2 (PTK2).

We used GeneCite software, a high throughput pathway analysis tool developed by our group, to identify pathways regulated by PA_17 _in PBMC [25]. A dramatic finding was that components of the cell adhesion pathway were up regulated by rPA_20 _in PBMC (Figure 8).

**Figure 9:**
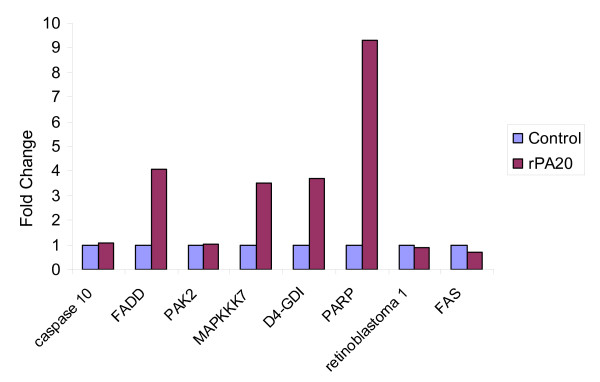
**Expression profile of genes related to the Fas pathway that were regulated by rPA_20 _in PBMC.** Cells were incubated with the rPA_20 _for 4 hrs. RNA samples were isolated and hybridized on the cDNA micorarray slides as detailed in materials and methods. Images were analyzed using GenePix 4.0 and data were analyzed using GeneSpring 7.0. Data were then analyzed using GeneCite to identify pathways regulated by the rPA_20_. These genes are caspase 10, Fas (TNFRSF6)-associated via death domain (FADD), p21 (CDKN1A)-activated kinase 2 (PAK2), mitogen-activated protein kinase kinase kinase 7 (MAPKKK7), Rho GDP dissociation inhibitor (GDI) alpha (D4-GDI), ADP-ribosyltransferase (NAD+; poly (ADP-ribose) polymerase), retinoblastoma 1 and tumor necrosis factor receptor superfamily, member 6 (FAS).

Another pathway found to be regulated was the Fas pathway. Figure 9 illustrates expression patterns of some of these components in cells treated with rPA_20_.

### Induction of apoptosis by rPA_20_

**Figure 10:**
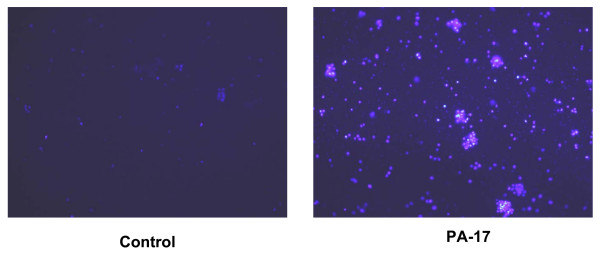
**Induction of apoptosis in PBMC with rPA_20_. **Cells were incubated with the rPA_20 _for 24 hours. Cells were then stained with HOECHST 33258 dye for 15 minutes. Cells with bright, fragmented, condensed nuclei were identified as apoptotic cells. This experiment was conducted at least 4 times (representative field shown here).

**Figure 11:**
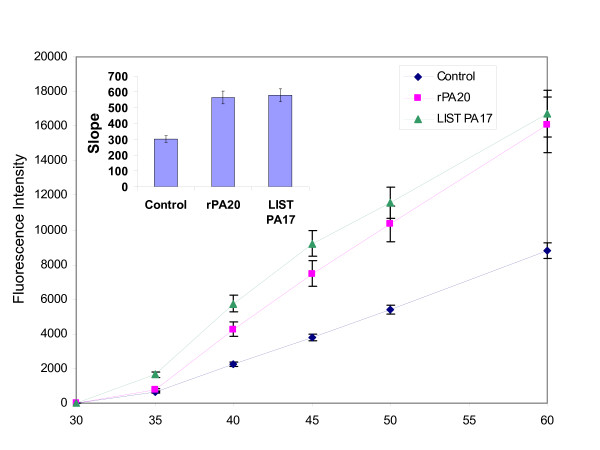
**LIST PA_17 _and rPA_20 _induce Caspase 3 enzymatic activity in PBMC. **Cells were exposed to LIST PA_17 _and to rPA_20 _separately and harvested at indicated time points. Cells were lysed and centrifuged. The mixture was incubated for 30 min and the fluorescence was measured using excitation/emission ~496/520 nm.

PBMC cells were stained with the DNA binding dye Hoechst 33258 to determine the number of apoptotic cells. When PBMC cells were incubated with rPA_20 _for 24 hours, the percentage of apoptotic cells was increased by more than 5-fold with respect to control cells (Figure 10). Similar effects were observed in cells incubated with the PA_17 _peptide. We have also studied the effect of PA_17 _and rPA_20 _on the caspase activity and found increased enzymatic activity of Caspase 3 in PBMC that were exposed to PA_17 _and rPA_20 _(Figure 11).

### Effect of rPA_20 _on CD38 cells

**Figure 12:**
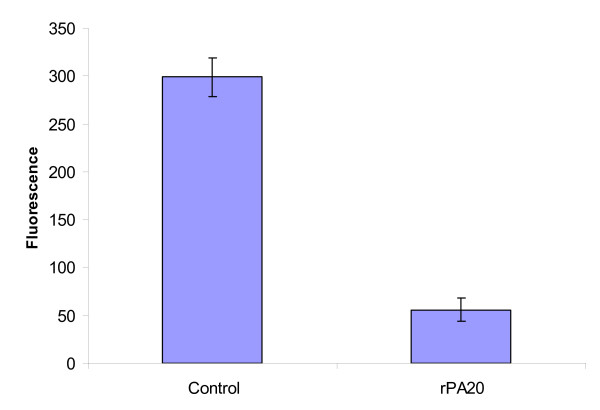
**Effect of rPA_20 _on the expression of CD38 in PBMCs: Cells were incubated with rPA20 for 16 hours.** Cells were then centrifuged and incubated with allophycocyanin (APC)-conjugated mouse anti-human CD38 monoclonal antibody. Samples were loaded onto a cell chip which determined cell associated fluorescence. Data are expressed as percent of CD38 fluorescent cell compared to total cell number (+/- standard deviation) and represent the results of three separate experiments performed in triplicate.

Microarray data analysis showed that CD38 transcription level was significantly down regulated in PBMC treated with rPA_20_. We carried out an antibody staining analysis of CD38 using the cell chip assay on the Bioanalyzer 2100 and found a decrease in CD38-associated fluorescence (Figure 12).

## Discussion and conclusion

It is widely accepted that *B. anthracis *toxins contribute to anthrax pathogenesis and to date, only the combination of the PA_63 _fragment in association with LF or EF have been described. PA is secreted by the bacterium as an 83 kDa protein which is rapidly cleaved in sera to PA_63 _and a remaining 20 kDa fragment. PA63 has been shown to form a heptamer that binds LF and forms a cellular pore via receptor mediated endocytosis to facilitate the entry of LF or EF into the host target cells. We have detected PA_20 _in the blood of infected animals, and to date no activity has been described for this PA fragment. In addition, PA83 has not been detected in the blood of B. anthracis infected animals. We have previously reported that PA83 is cleaved by a calcium dependent plasma protease to PA63 which forms oligomeric complexes with other PA63 and LF (and possibly EF), to result in the respective anthrax toxins [17].

Although the 20 kDa fragment of PA is commercially available (LIST Biologicals), our recent studies showed that the procedure used resulted in a trypsin resistant 17 rather than a 20 kDa fragment (mass spectroscopy analysis). In contrast, *in vivo*, the fragment produced is 20 kDa. Although we used the rPA_20 _for the studies reported here, we showed that the PA_17 _has similar activities to the rPA_20 _on PBMC. To understand the role that PA_20 _plays in the pathogenesis of *B. anthracis*, we carried out a global genomic analysis of the effect of rPA_20 _on PBMC *in vitro*. Some of the genes found to be regulated by rPA_20 _are related to apoptosis and cell growth. In addition, several cytokine related genes were up regulated by rPA_20_. This observation is consistent with the reported effect of *B. anthracisin vitro *and *in vivo *[26]. A recent publication using modeling approaches identified a 14 kDa sequence (14–150) as a critically conserved domain in bacterial toxins, adhesins other crucial molecules for biological activity [27]. It should be noted that this study was a survey of sequences using bioinformatics and modeling and did not utilize an actual fragment from PA.

Another interesting observation is the effect of rPA_20 _on the expression of CD38 that was significantly decreased by rPA_20_. CD38 is a type II integral membrane receptor and adhesion molecule [28] and serves as a cytotoxic triggering molecule on natural killer cells [29].

Components of the Fas pathway were up regulated in PBMC treated with rPA_20_. We have also found that rPA_20 _increased the caspase-3 activity in PBMC cells. Thus we expected that rPA_20 _may induce apoptosis in these cells.

The accepted model of PA_63_/LF complex and the deleterious effects attributed to this complex have not taken into consideration any possible effects of the rPA_20 _which is released when the complex is formed. Here we propose that rPA_20 _may be responsible for some of the effects previously ascribed to PA_63_/LF on host cells. A recent publication described the association of rPA_20 _with LF [30]; it is therefore possible that PA_20 _has additional functions which may contribute to pathogenesis and should be considered.

## Conclusion

The 20 kDa component of the protective antigen may play a role in the pathogenesis of *Bacillus anthracis *and should be studied in more details.

## Abbreviations

PA: protective antigen; LF: Lethal factor; EF: Edema factor; PBMC: Peripheral blood momonuclear cells; TNF-α: Tumor necrosis factor; IL: Interleukin

## Competing interests

The authors declare that they have no competing interests.

## Authors' contributions

RH drafted the manuscript, performed the genomic analysis, data mining and the apoptosis studies. WJR carried out the production, purification and characterization of the rPA_20_. TGA carried out the exposures to *B. anthracis*. MJ conceived of the study, and participated in its design and coordination. JWE participated in the design and coordination on the study. All authors read and approved the final manuscript.

## Pre-publication history

The pre-publication history for this paper can be accessed here:


